# Implicit motor sequence learning using three-dimensional reaching movements with the non-dominant left arm

**DOI:** 10.1007/s00221-024-06934-4

**Published:** 2024-10-08

**Authors:** Charles R. Smith, Jessica F. Baird, Joelle Buitendorp, Hannah Horton, Macie Watkins, Jill C. Stewart

**Affiliations:** 1 Department of Exercise Science, Arnold School of Public Health, University of South Carolina, Columbia, SC USA; 2 Johns Hopkins Trial Innovation Center, Johns Hopkins School of Medicine, Baltimore, MD, USA

**Keywords:** Motor learning, Implicit sequence learning, Reaching, Dominance

## Abstract

Interlimb differences in reach control could impact the learning of a motor sequence that requires whole-arm movements. The purpose of this study was to investigate the learning of an implicit, 3-dimensional whole-arm sequence task with the non-dominant left arm compared to the dominant right arm. Thirty-one right-hand dominant adults completed two consecutive days of practice of a motor sequence task presented in a virtual environment with either their dominant right or non-dominant left arm. Targets were presented one-at-a-time alternating between Random and Repeated sequences. Task performance was indicated by the time to complete the sequence (response time), and kinematic measures (hand path distance, peak velocity) were used to examine how movements changed over time. While the Left Arm group was slower than the Right Arm group at baseline, both groups significantly improved response time with practice with the Left Arm group demonstrating greater gains. The Left Arm group improved performance by decreasing hand path distance (straighter path to targets) while the Right Arm group improved performance through a smaller decrease in hand path distance combined with increasing peak velocity. Gains made during practice on Day 1 were retained on Day 2 for both groups. Overall, individuals reaching with the non-dominant left arm learned the whole-arm motor sequence task but did so through a different strategy than individuals reaching with the dominant right arm. The strategy adopted for the learning of movement sequences that require whole-arm movements may be impacted by differences in reach control between the nondominant and dominant arms.

**Supplementary Information:**

The online version contains supplementary material available at 10.1007/s00221-024-06934-4.

## Introduction

The learning or relearning of functional tasks and skills is important for effective execution of tasks in daily life. Implicit motor learning is defined as the acquisition of a motor skill through repetitive practice of the task without explicit knowledge of how the task is completed (Seger 1994; Sun et al. 2005; Dale et al. 2012) and elicits robust changes in performance (Lee and Vakoch 1996; Ghilardi et al. 2009; Steenbergen et al. 2010; Sanchez and Reber 2013; Kal et al. 2018; van Es and Knapen 2019). Implicit learning has often been investigated using sequence-based tasks that require finger movements (Nissen and Bullemer 1987; Boyd and Winstein 2003, 2006; Lin et al. 2011; Ariani and Diedrichsen 2019; Yokoi and Diedrichsen 2019) or goal-directed movements in two-dimensional (2D) space (Seidler 2006; Ghilardi et al. 2009; Moisello et al. 2009; Meehan et al. 2011; Wadden et al. 2017; de Kleijn et al. 2018). However, most functional tasks of daily living occur in unconstrained, three-dimensional (3D) environments. These movements require simultaneous, coordinated movement of multiple joints while moving against gravity thereby making them more demanding and complex than the tasks commonly used to examine learning (Sande de Souza et al. 2009; Ambike and Schmiedeler 2013; Dounskaia and Wang 2014; d’Avella et al. 2015; Schaffer and Sainburg 2017). We previously investigated implicit sequence learning using a whole-arm 3D reaching task (serial target task) (Baird and Stewart 2018). Importantly, this task paradigm allows the investigation of spatial (hand path) and speed (velocity) features of arm control over practice in addition to overall performance (response time). In this previous work, however, learning was investigated only in the dominant right arm which means its results may not apply to learning in the non-dominant left arm.

Previous studies have shown interlimb differences in reach control between the dominant and non-dominant arms in right-hand dominant individuals. Reaches with the dominant right arm tend to show relatively low initial direction error and straight hand paths, which indicates a high degree of inter-joint coordination between the shoulder and elbow and greater reliance on feedforward control (Sainburg and Kalakanis 2000; Bagesteiro and Sainburg 2002; Tomlinson and Sainburg 2012; Mutha et al. 2013). In contrast, targeted reaches with the non-dominant left arm have consistently shown high initial direction errors and curved hand paths, indicating poorer inter-joint coordination, but lower final position errors, indicating better end-point accuracy and greater reliance on feedback control (Sainburg and Kalakanis 2000; Bagesteiro and Sainburg 2002; Przybyla et al. 2012; Tomlinson and Sainburg 2012; Mutha et al. 2013). However, recent studies have indicated that lateralized motor behavior persists in the absence of sensory feedback influences, suggesting non-dominant arm control may not be solely reliant on feedback control mechanisms (Jayasinghe et al. 2020, 2021). Studies specifically examining unsupported, 3D targeted reaching have shown either higher (Schaffer and Sainburg 2017) or similar final position errors (Tomlinson and Sainburg 2012) for reaches with the non-dominant left arm compared to the dominant right arm suggesting possible differences in end-point control based on task conditions. Recent examination of dominant versus non-dominant reach control in a deafferented patient relative to controls indicated that these differences in control between the two arms and in different conditions may be related to how proprioceptive information is utilized by each arm when reaching (Jayasinghe et al. 2020, 2021). These interlimb differences in reach control between the dominant and non-dominant arms may impact the manner by which a whole-arm movement sequence is learned.

Investigations of sequence learning in the non-dominant left arm have been limited and generally involved simple finger-pressing paradigms (Nissen and Bullemer 1987; Boyd and Winstein 2006; Lin et al. 2011; Ariani and Diedrichsen 2019; Yokoi and Diedrichsen 2019). Tasks which involve more complex whole-arm movements allow for the examination of how differences in control between the arms may influence learning (Grafton et al. 2002; Haaland et al. 2004; Verwey and Clegg 2005). Studies using two-dimensional targeted reaching movements showed differences in the learning of reach movements between the dominant and non-dominant limbs which further emphasized the use of feedforward and feedback control strategies, respectively (Sainburg and Wang 2002; Criscimagna-Hemminger et al. 2003; Buchanan 2004; Buchanan et al. 2007; Duff and Sainburg 2007; Mutha et al. 2012, 2013; Stockinger et al. 2015; Bagesteiro et al. 2021). However, sequence learning during 3D whole-arm reaching movements with the non-dominant versus the dominant limb has not been examined.

The purpose of this study was to investigate the learning of an implicit, 3D whole-arm sequence task with the non-dominant left arm compared to the dominant right arm. It was hypothesized that the non-dominant left arm would show slower response times which would correspond with longer hand paths indicating greater hand path curvature than the dominant right arm at baseline consistent with previous studies on the control of reaching (Sainburg and Kalakanis 2000; Bagesteiro and Sainburg 2002; Przybyla et al. 2012; Tomlinson and Sainburg 2012; Mutha et al. 2013; Schaffer and Sainburg 2017; Jayasinghe et al. 2021). However, it was hypothesized that response times would improve with practice in both arms, demonstrating learning of the motor sequence. Finally, we explored whether the two arms differed in the approach taken to improve response time (spatial and/or speed) to examine if the left arm improved overall performance through general gains in both spatial and speed control or through gains in control in areas of deficit at baseline (i.e. spatial control).

## Methods

### Participants

Thirty-one non-disabled, neurologically intact adults completed the motor sequence task. To be eligible for participation, individuals had to be right-hand dominant as determined by the Edinburgh Handedness Questionnaire (EHI) (Oldfield 1971), between 18 and 40 years of age, have no current or recent neurological symptoms as determined by a general symptom checklist, and no reported pain in the upper extremities. Sixteen participants (7 female, 26.0 ± 4.6 yrs, EHI laterality quotient = 69.2 ± 28.1) were recruited for this study and completed the serial target task with their non-dominant left arm. Data from fifteen participants (6 female, 23.5 ± 3.7 yrs, EHI laterality quotient = 75.8 ± 27.5) who completed the task using their dominant right arm were previously collected and results reported in Baird and Stewart (2018); this previously collected data was used for the purposes of comparison between the dominant right and non-dominant left arms. All participants provided written informed consent prior to enrollment in the study. The study was conducted in accordance with the Declaration of Helsinki, and all aspects of the study were approved by the Institutional Review Board (IRB) at the University of South Carolina.

### Experimental task

The serial target task completed in this study was described in detail in Baird and Stewart (2018). Briefly, participants sat facing a virtual display (Innovative Sport Training Inc., Chicago, IL) where the task was projected down into the workspace directly in front of them. The participants wore stereoscopic glasses to allow for 3D visualization of the targets (28 mm red sphere) that were all in the same Z plane. An electromagnetic marker placed on the index finger was used to both indicate position in the virtual display (cursor, 25 mm white sphere) and collect position data throughout movement. Participants were instructed to reach to the projected target as quickly and accurately as possible; all movements were 3D and unsupported. Once the center of the cursor was within 5 mm of the center of the target for ≥ 500 msec, the target was considered “hit” and would disappear as the next target appeared. Online visual feedback of the cursor and target position was present throughout.

**Fig. 1:**
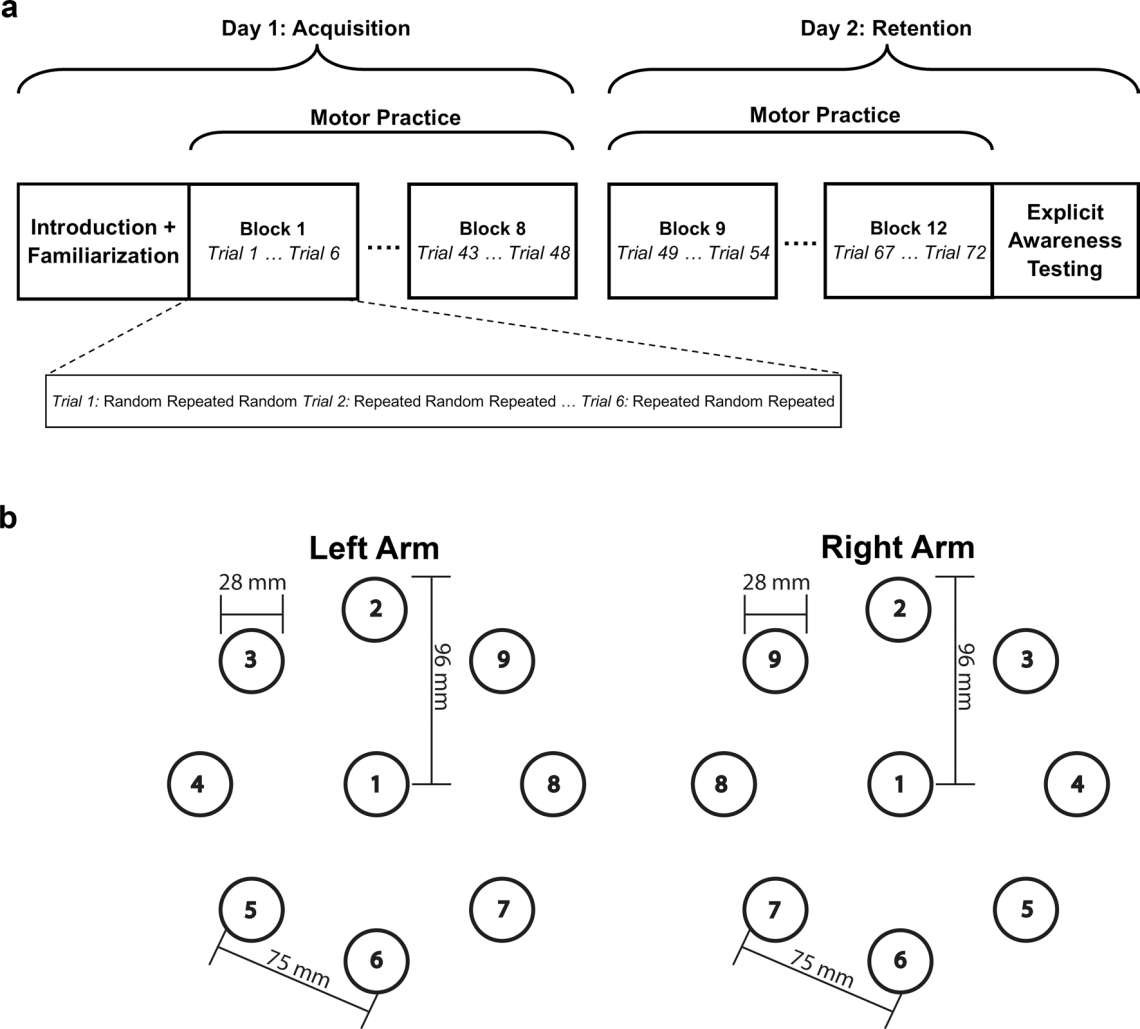
*Experimental Setup*. **a** Experimental design. Participants completed 8 blocks of practice on Day 1 and 4 blocks of practice on Day 2 (18 sequence repetitions per block alternating between Random and Repeated). **b** Overhead view of the circular target array for the Left and Right arms. All targets were presented in the same Z plane (height). The Repeated sequence consisted of targets 1–8 – 6–5 – 9–4 – 8–2; mm = millimeters

The serial target task was comprised of two sequence conditions: repeated and random. Each sequence consisted of eight targets and were controlled for difficulty by matching the total straight-line inter-target distance (93.8 cm). For the Left Arm group, the target array was mirrored such that inter-target movements were in the same direction relative to the person as they were for the Right Arm group (Fig. 1). Individual movements between any two targets were assigned an Index of Difficulty (ID) value based on Fitts’ Law (*ID* = *log*_2_(2*A/W*) where A = target amplitude or distance and W = target width or diameter) (Fitts and Peterson 1964; Fitts 1966; Meehan et al. 2011). Calculated values of each ID were 2.42, 2.78, 3.28, 3.66, and 3.78 in increasing order based on inter-target distance. To simplify, targets were assigned an ID value between 1 and 5 with 1 being the shortest movement (ID = 2.42) and 5 being the longest movement (ID = 3.78). Each sequence was assigned targets consisting of the same ID levels such that every eight-target sequence was comprised of one movement at ID levels 1 and 4 and two movements at ID levels 2, 3, and 5. The repeated sequence (1–8 – 6–5 – 9–4 – 8–2) was the same across all trials. Random sequences were comprised of pseudorandomly assigned targets such that overall difficulty (total inter-target distance) was the same as the repeated sequence.

All data were collected using the MotionMonitor system (Innovative Sport Training Inc., Chicago, IL). An electromagnetic sensor (Flock of Birds, Ascension Technology Corp, Shelburne, VT) was attached to the nailbed of the index finger of the arm used to complete the sequence. Positional data was sampled at a rate of 120 Hz, and data were analyzed using a customized script in MATLAB (Mathworks Inc., Natick, MA). Consistent with previous studies using a similar task (Brodie et al. 2014a, b; Baird and Stewart 2018), total time to complete an eight-target sequence (response time) was the primary measure of task performance. To determine how performance changed over time, both spatial and speed kinematic variables were evaluated. The spatial kinematic variable was the total length of the hand path (sum of total distance moved) when completing a sequence whereby a shorter total movement distance indicated straighter hand paths between targets. The speed kinematic variable was peak velocity which was calculated by dividing the change in the 3D linear movement trajectory by the change in time (Winter 2005). The peak of velocity was extracted from each movement between two targets and averaged across each eight-target sequence. A higher peak velocity indicated faster reaching movements.

### Experimental procedure

Participants completed the 3D reach task over two consecutive days separated by 24 ± 2 h (Fig. 1). First, participants were introduced to the task environment by reaching to move the cursor representing hand position toward each target in the circular array. Next, participants completed a series of 24 targeted reaches to become familiar with the presentation of targets. On Day 1 (Acquisition), individuals then practiced 144 total sequences in alternating random-repeated sequence order presented in 8 Blocks of 6 trials each with each trial containing three sequence repetitions (Fig. 1). Ten seconds of rest was provided after every trial (three sequences) and one minute rest after every Block (18 sequences). Participants were not informed of the presence of the repeated sequence during practice. All participants returned on Day 2 (Retention) for retention testing whereby they completed an additional 72 alternating random-repeated sequences. All other procedures were identical to Day 1.

After completion of the retention test on Day 2, explicit awareness of the repeated sequence was assessed. All participants viewed six explicit awareness tests containing three eight-target sequences presented in the virtual environment. After each test, the participant was asked if the repeated sequence was present and, if so, which of the eight-target sequences contained the repeated sequence (beginning, middle or end). Three of the six tests contained the repeated sequence (positive test) while the remaining three tests contained a random sequence (negative test). Participants were classified as “aware” of the repeated sequence if they correctly identified the repeated sequence in two out of the three positive tests while also correctly identifying two out of the three negative tests.

### Statistical analysis

All statistical analyses were completed using SPSS v.25 (IBM Corp., Armonk, NY). Data from each sequence type (Random, Repeated) were averaged into blocks of nine sequences for analysis (Day 1, Acquisition = 8 blocks of 9 sequences; Day 2, Retention = 4 blocks of 9 sequences). To examine differences between groups at Baseline, response time and kinematic outcomes (total hand path distance, peak velocity) in Block 1 were analyzed using a mixed model analysis of variance (ANOVA) with a between-subject effect for Group (Right Arm, Left Arm) and within-subject effect for Sequence type (Random, Repeated). Changes in response time and the kinematic outcomes across Day 1 (Acquisition) were assessed using a 2 × 2 × 8 mixed model ANOVA with a between-subject factor for Group (Right Arm, Left Arm) and within-subject factors for Sequence type (Random, Repeated) and Block (Day 1 Blocks 1–8). Significant interactions were followed up with t-tests to examine the locus of the change in the Acquisition phase (from Block 1 to Block 8). Retention was examined as the change between the end of Day 1 (Block 8) and the start of Day 2 (Block 9) using a 2 × 2 × 2 mixed model ANOVA with a between-subject factor for Group (Right Arm, Left Arm) and within-subject factors for Sequence type (Random, Repeated) and Time (Block 8, Block 9). An improvement in outcomes from the end of Day 1 to the start of Day 2 was defined as consolidation while a worsening in outcomes from the end of Day 1 to the start of Day 2 was defined as forgetting. Significant interactions were followed up with t-tests to examine the locus of the change. When the assumption of sphericity was violated, the Greenhouse-Geisser corrected p-values were used. All analyses were completed with significance set at *p* < 0.05. Partial eta squared (ƞ^2^) was used to estimate the effect sizes of main effects and interactions (ƞ^2^ of 0.01–0.059 = small effect; ƞ^2^ of 0.06–0.139 = medium effect; ƞ^2^ ≥ 0.140 = large effect) while effect sizes for follow-up analyses were assessed using Cohen’s d (d of 0.01–0.19 = very small effect; d of 0.20–0.49 = small effect; d of 0.50–0.79 = medium effect; d of 0.80–1.19 = large effect; d ≥ 1.20 = very large effect) (Cohen 1988).

## Results

### Baseline performance

**Fig. 2:**
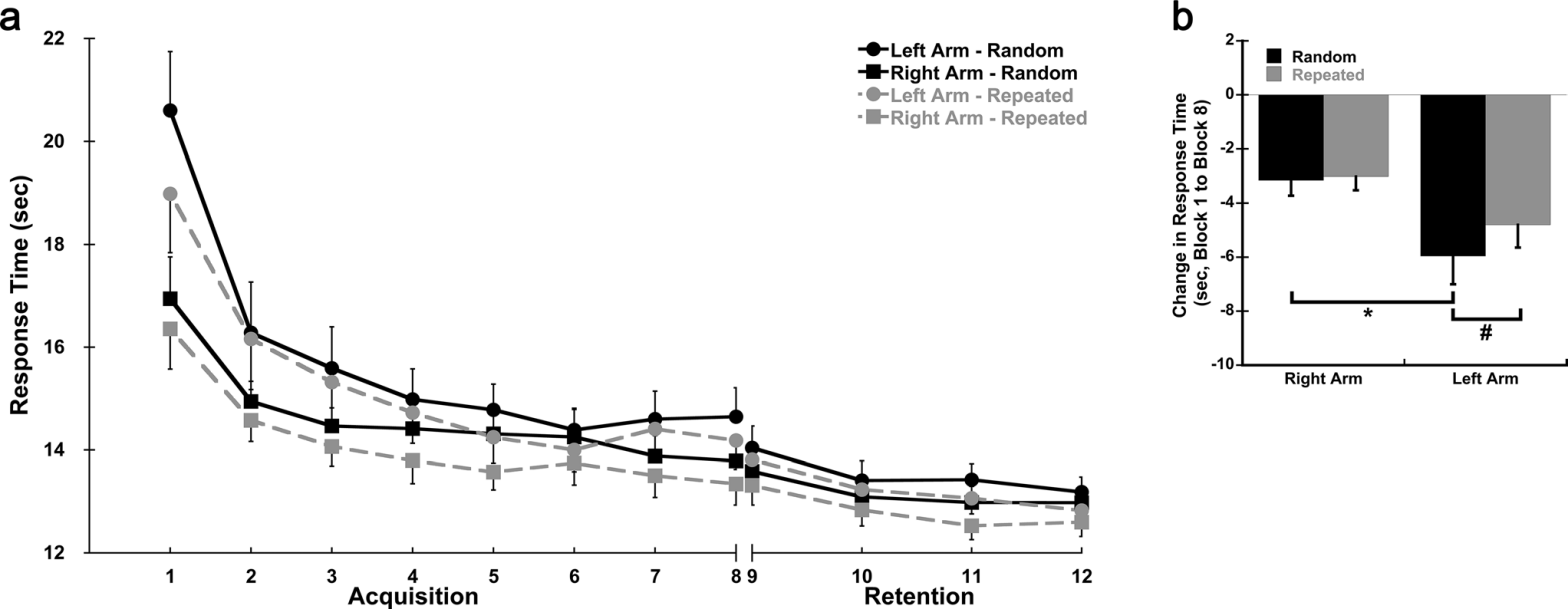
*Response Time over Practice*. **a** Average response time by block for the Random (solid lines) and Repeated (dashed lines) sequences for each group. Blocks 1–8 were completed on Day 1 (Acquisition). Blocks 9–12 were completed on Day 2 (Retention). Each block consisted of 9 sequences. **b** Average change in response time (Block 8 – Block1) for the Random and Repeated sequences in both the dominant right and non-dominant left arm groups on Day 1 (Acquisition); sec = seconds; * = significant difference between arms; # = significant difference between sequence type; data presented as mean ± SEM

**Fig. 3:**
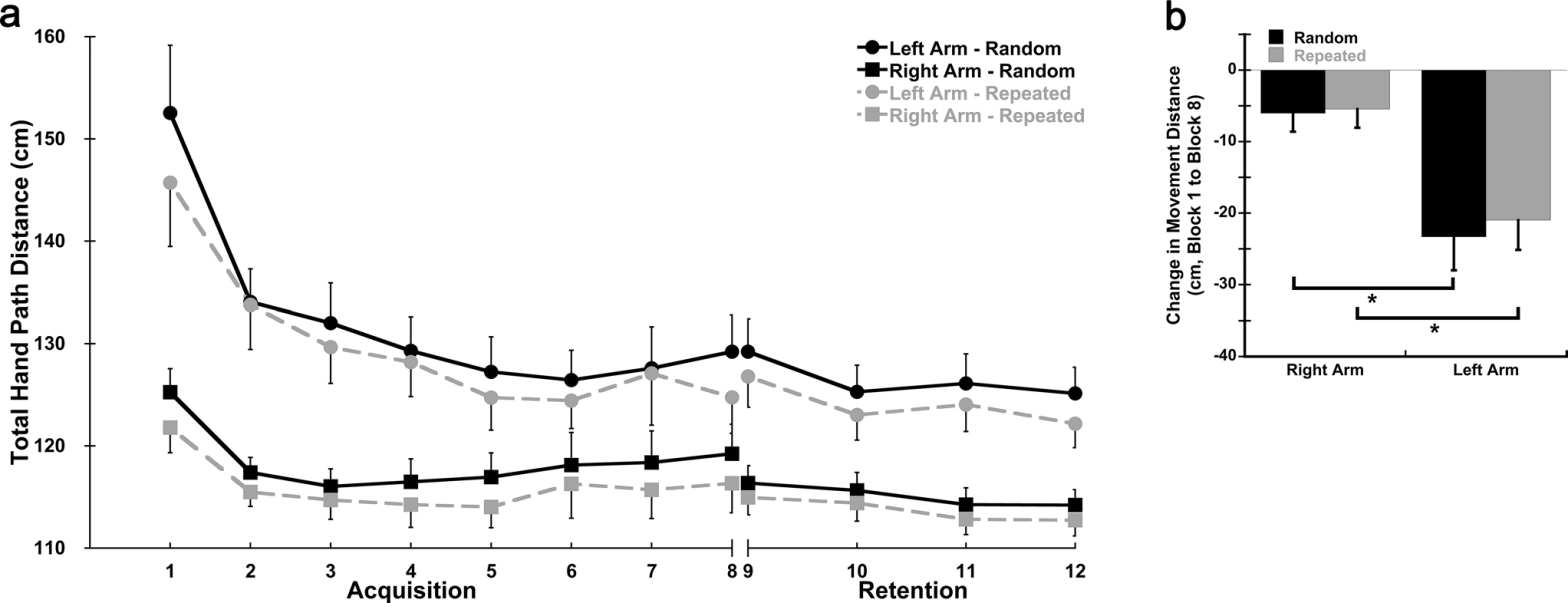
*Hand Path over Practice*. **a** Average total hand path distance by block for the Random (solid lines) and Repeated (dashed lines) sequences for each group. Blocks 1–8 were completed on Day 1 (Acquisition). Blocks 9–12 were completed on Day 2 (Retention). Each block consisted of 9 sequences. **b** Average change in total hand path distance (Block 8 – Block1) for the Random and Repeated sequences in both the dominant right and non-dominant left arm groups on Day 1 (Acquisition); total hand path distance = total distance of hand movement during completion of the sequence; cm = centimeters; * = significant difference between arms; data presented as mean ± SEM

**Fig. 4:**
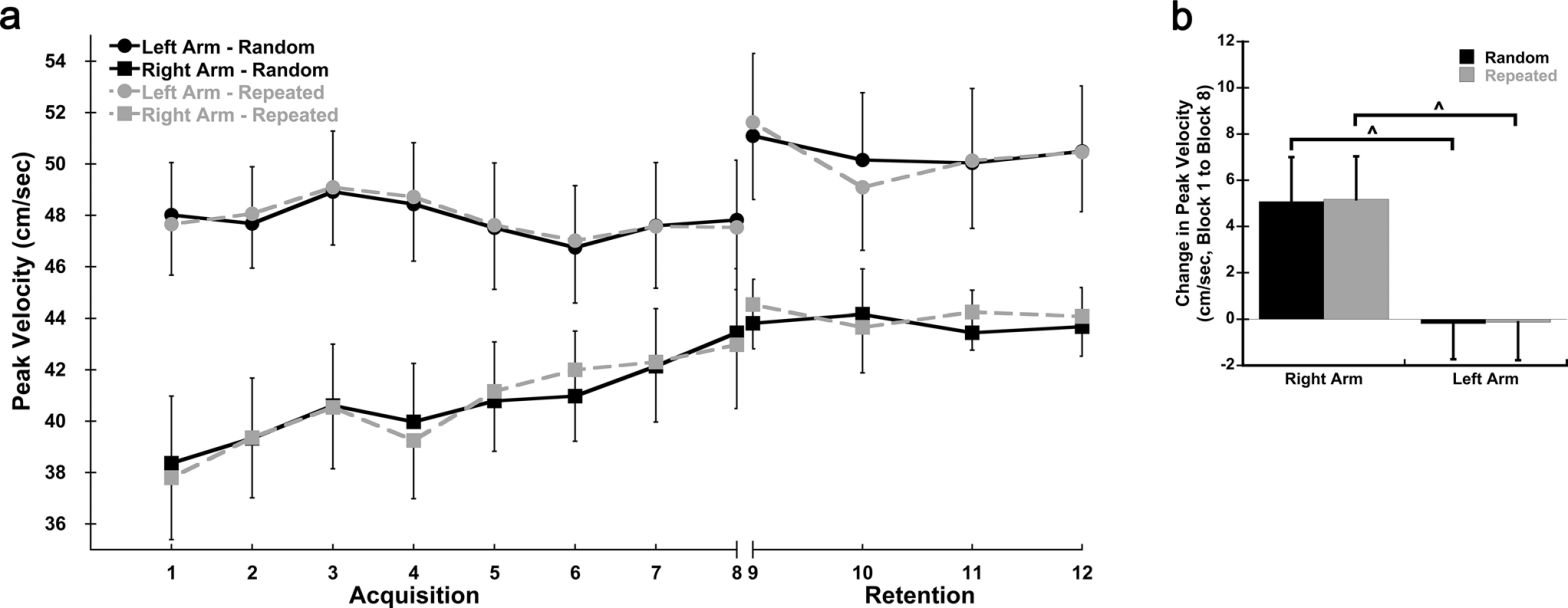
*Peak Velocity over Practice*. **a** Average peak velocity by block for the Random (solid lines) and Repeated (dashed lines) sequences for each group. Blocks 1–8 were completed on Day 1 (Acquisition). Blocks 9–12 were completed on Day 2 (Retention). Each block consisted of 9 sequences. **b** Average change in peak velocity (Block 8 – Block1) for the Random and Repeated sequences in both the dominant right and non-dominant left arm groups on Day 1 (Acquisition); cm = centimeters; sec = seconds; ^ =significant difference between arms; data presented as mean ± SEM

Response time, total hand path distance, and peak velocity in the first block of practice on Day 1 were used to assess baseline performance. There was a significant Sequence X Group interaction for response time in Block 1 (*p* = 0.022; η^2^ = 0.169; Fig. 2a, Supplemental Table 1). The Right Arm group had significantly lower response times than the Left arm group for the Random (*p* = 0.032; d = 0.802) but not the Repeated sequence (*p* = 0.069; d = 0.673). There was also a sequence-specific effect present whereby the Repeated sequence had overall lower response times than the Random sequence during Block 1 (*p* < 0.001; η^2^ = 0.482). Hand path distance differed between groups in Block 1 (Fig. 3a); the Right Arm group had shorter hand path distances than the Left Arm group regardless of sequence (*p* = 0.001; η^2^ = 0.316). A sequence-specific effect was observed for total hand path distance in Block 1 whereby the Repeated sequence had shorter hand path distances than the Random sequence (*p* < 0.001; η^2^ = 0.560). Peak velocity also differed between groups in Block 1 (Fig. 4a); the Left Arm group had higher peak velocities than the Right Arm group (*p* = 0.005; η^2^ = 0.244). However, there were no differences in peak velocity between the Random and Repeated sequences in Block 1 (*p* = 0.129; η^2^ = 0.078). No additional significant interactions were observed.

Given that Block 1 included nine sequence repetitions in each condition and that practice improvements may have been evident early, an exploratory analysis of the first practice repetition for each sequence was conducted to examine initial response to the task. In this first practice repetition, there was no difference between sequence types for response time (*p* = 0.434; η^2^ = 0.026), peak velocity (*p* = 0.824; η^2^ = 0.002), or hand path distance (*p* = 0.546; η^2^ = 0.015) (Supplemental Fig. 1), suggesting that differences between sequence type in Block 1 were due to early practice effects. For this first repetition, compared to the Left Arm group, the Right Arm group had lower response times (*p* = 0.039; η^2^ = 0.166; mean difference = 5.71 s) and shorter total hand path distance (*p* = 0.004; η^2^ = 0.292; mean difference = 42.9 cm), consistent with the analysis of Block 1. While peak velocity was lower in the Right Arm group compared to the Left Arm group similar to Block 1, the difference was not statistically significant (peak velocity: *p* = 0.085; η^2^ = 0.119; mean difference = 7.62 cm/sec). Further analysis of Block 1 is provided in the Supplemental Results.

### Acquisition

During Acquisition, there were significant Block X Group (*p* = 0.026; η^2^ = 0.126) and Block X Sequence (*p* = 0.008; η^2^ = 0.119) interactions for response time indicating there were differences in improvement between the two arms and sequences over practice (Fig. 2a, Supplemental Table 2). Follow-up comparison on the change in response time from Block 1 to Block 8 (Fig. 2b) showed that the Left Arm group saw greater improvements in response time for both the Random (Left = -5.95 ± 1.05 s, Right = -3.15 ± 0.57 s; *p* = 0.028; d = 0.829) and Repeated sequences (Left = -4.80 ± 0.84 s, Right = -3.01 ± 0.51 s; *p* = 0.085; d = 0.642) than the Right Arm group, but only the difference between the arms for the Random sequence was statistically significant. The Left Arm group also saw greater improvements in the Random sequence compared to the Repeated sequence (*p* = 0.014; d = 0.696); there were no differences between the sequence types in the Right Arm group (*p* = 0.419; d = 0.215).

There were also significant Block X Group (*p* = 0.002; η^2^ = 0.191) and Block X Sequence (*p* = 0.002; η^2^ = 0.137) interactions for total hand path distance indicating there were differences in improvement between the two groups and sequences over practice (Fig. 3a). Follow-up comparison on the change from Block 1 to Block 8 (Fig. 3b) showed that the Left Arm group had significantly greater decreases in total hand path distance compared to the Right Arm group regardless of sequence (*p* < 0.005; Random: Left = -23.31 ± 4.69 cm, Right = -6.03 ± 2.58 cm, d = 1.139; Repeated: Left = -20.98 ± 4.15 cm, Right = -5.43 ± 2.62 cm, d = 1.121).

A significant Block X Group interaction was seen for peak velocity (*p* = 0.033; η^2^ = 0.105) indicating the two groups had different changes in velocity over practice (Fig. 4a). A follow-up comparison on the change in peak velocity from Block 1 to Block 8 (Fig. 4b) showed that while peak velocity in the Left Arm group remained relatively constant on Day 1, peak velocity in the Right Arm group increased with practice (*p* < 0.05; Random: Left = -0.19 ± 1.54 cm/sec, Right = + 5.08 ± 1.92 cm/sec, d = 0.774; Repeated: Left = -0.13 ± 1.64 cm/sec, Right = + 5.18 ± 1.86 cm/sec, d = 0.771). No significant difference in peak velocity was found between sequences in either group (*p* > 0.720; Left η^2^ = 0.003, Right η^2^ = 0.009).

### Retention

Response time did not significantly change from the end of Day 1 to the start of Day 2 (*p* = 0.105; η^2^ = 0.088) indicating that overall performance was retained and no forgetting occurred (Fig. 2, Supplemental Fig. 2, Supplemental Table 3). The sequence-specific effects from Day 1 (Acquisition) remained whereby response times were lower for the Repeated sequence than the Random sequence (*p* < 0.001; η^2^ = 0.488). Response times did not differ between the Right and Left Arm groups (*p* = 0.267; η^2^ = 0.042). No significant interactions were found.

For total hand path distance, a significant Time X Sequence interaction (*p* = 0.002; η^2^ = 0.276) was followed-up with a paired t-test comparing the change from Block 8 to Block 9 between sequences. Between these blocks, hand path distance continued to decrease for the Random sequence (consolidation) while hand path distance for the Repeated sequence remained relatively constant (Random = -1.39 ± 1.4 cm, Repeated = + 0.39 ± 1.4 cm; *p* = 0.002; d = 0.607) indicating no forgetting and retention from the end of Day 1 to the start of Day 2 (Fig. 3, Supplemental Fig. 2). The sequence-specific and group effects from Day 1 (Acquisition) remained whereby the hand paths were shorter for the Repeated sequence than the Random sequence (*p* < 0.001; η^2^ = 0.700) and the Right Arm group had shorter hand paths than the Left Arm group (*p* = 0.009; η^2^ = 0.210).

Peak velocity also had a significant Time X Sequence interaction (*p* = 0.002; η^2^ = 0.282). Follow-up t-tests comparing the change from Block 8 to Block 9 between sequences indicated that movement speed was retained. While the peak velocities for both sequences increased (consolidation), the Repeated sequences increased to a greater degree than those of the Random sequences from the end of Day 1 to the start of Day 2 (Random = + 1.87 ± 0.97 cm/sec, Repeated = + 2.87 ± 0.99 cm/sec; *p* = 0.002; d = 0.609) (Fig. 4, Supplemental Fig. 2). While the Left Arm group showed an increase in peak velocity from Block 8 to Block 9 that was not as evident in the Right Arm group, there was no main effect of Group (*p* = 0.139; η^2^ = 0.074) or Time X Group interaction effect (*p* = 0.165; η^2^ = 0.065) indicating that both the dominant Right and non-dominant Left arm had similar peak velocities from the end of Acquisition to the start of Retention.

### Explicit awareness

In the Right Arm group, six participants were aware of the Repeated sequence. Per a previous analysis, changes in response time with practice did not differ between those who recognized the presence of a sequence and those who did not, suggesting that awareness of the Repeated sequence did not influence overall task performance (Baird and Stewart 2018). In the Left Arm group, no participants were aware of the repeated sequence.

## Discussion

The purpose of this study was to investigate the learning of an implicit, 3D whole-arm sequence task with the non-dominant left arm compared to the dominant right arm. While the non-dominant Left Arm group started off with slower response times than the dominant Right Arm group at baseline, the Left Arm group had significantly greater improvements in response time with practice, especially in the Random sequence. Faster response times for the Left Arm group were primarily achieved through improvements in spatial control (shorter hand paths) while the Right Arm group improved in both spatial control (shorter hand paths) and movement speed (increased peak velocity). Interestingly, the Left Arm group had consistently higher peak velocities than the Right Arm group across practice. Overall, these results show that while both the non-dominant Left and dominant Right Arm groups effectively learned the motor sequence task, the two arms achieved improvements by utilizing different strategies.

At baseline, the Left Arm group showed slower response times compared to the Right Arm group. The initial difference in response time between groups could be attributed at least in part to the non-dominant left arm being less skilled at complex motor sequence tasks than the dominant right arm (Haaland et al. 2004). However, Block 1 differences in task performance could also be attributed to the initial differences in spatial control and movement speed. Consistent with previous studies, the Left Arm group had poorer spatial control than the Right Arm group, as seen by longer hand paths which may be attributed to differences in inter-joint coordination (Sainburg and Kalakanis 2000; Sainburg and Wang 2002; Sainburg and Schaefer 2004). Previous studies have shown that in well-coordinated arm movements, the motor system employs the simplest pattern possible whereby either the shoulder or elbow joint initiates movement and exerts interaction torques that move the other, subordinate joint (Dounskaia 2005; Gritsenko et al. 2011; Ambike and Schmiedeler 2013; Dounskaia et al. 2020). According to the dynamic dominance hypothesis, the non-dominant left arm is less skilled at coordinating multi-joint movements compared to the dominant right arm resulting in less efficient interactions between the shoulder and elbow joints (Sainburg and Kalakanis 2000; Bagesteiro and Sainburg 2002; Sainburg and Schaefer 2004; Sainburg 2005; Tomlinson and Sainburg 2012; Schaffer and Sainburg 2017). In the current study, this challenge with the coordination of multi-joint reaches in the non-dominant left arm corresponded to longer, more curved hand paths.

Throughout practice, the Left Arm group had faster movement velocities than the Right arm group. Because the motor system attempts to use the simplest pattern possible to execute a complex task (Dounskaia et al. 2020), it may be that the non-dominant left arm utilized greater speeds to compensate for poorer shoulder-elbow coordination patterns. However, some studies which examined reach control and learning in both the dominant and non-dominant arms did not show differences in velocity between the two arms (Sainburg and Kalakanis 2000; Bagesteiro and Sainburg 2002; Sainburg and Schaefer 2004; Goble et al. 2006; Schaffer and Sainburg 2017; Dexheimer and Sainburg 2021). Of note, while endpoint accuracy was emphasized and measured in these studies, it was not imperative for the completion and progression of the task trials as it was in the present study. Another study that used a reach-to-grasp task that required participants to successfully grasp the object (i.e., required accuracy demands) found faster peak velocities during reaching with the left arm compared to the right arm in both healthy, right-handed controls and individuals with left hemisphere stroke. (Kantak et al. 2020). Together with the results of the current study (which included accuracy demands), this suggests that the control of movements using the non-dominant left arm may differ when end-point accuracy is required compared to when it is not.

Despite their initial differences, both groups learned the sequence task as demonstrated by improvements in response time over practice and a lack of forgetting from the end of Day 1 to the start of Day 2. While improved task performance was expected based upon previous studies examining sequence learning in dominant, right arm movements (Seidler 2006; Ghilardi et al. 2009; Moisello et al. 2009; Meehan et al. 2011; Wadden et al. 2017; de Kleijn et al. 2018), this study shows that the non-dominant left arm improved performance on the sequence task using a different approach than the dominant right arm. Specifically, the non-dominant Left Arm group saw greater improvements in hand path distance (i.e., greater decrease in distance traveled) than the dominant Right Arm group. Since the non-dominant left arm is less skilled at generating coordinated movements and straight hand paths compared to the dominant right arm (Sainburg and Kalakanis 2000; Sainburg and Schaefer 2004; Sainburg 2005; Goble et al. 2006; Wang and Sainburg 2007), repeated practice of targeted reaching actions may have induced improvements in the left arm’s coordination ability leading to straighter, shorter hand paths. The Left Arm group did not show increases in velocity over practice suggesting that improvements in response time in this group could be attributed primarily to improvements in spatial control (hand path). This would indicate that the Left Arm group learned predominantly through improvements in shoulder-elbow coordination while maintaining the initial fast movement velocities throughout. In contrast, the Right Arm group exhibited improvements in both spatial control (hand path) and speed (peak velocity) over practice. The dominant right arm is effective at generating simple, well-coordinated joint patterns that yield relatively straight hand paths compared to the non-dominant left arm (Sainburg and Kalakanis 2000; Bagesteiro and Sainburg 2002; Sainburg and Schaefer 2004; Sainburg 2005; Schaffer and Sainburg 2017). Therefore, the dominant right arm may learn a whole-arm sequential target task by both refining that already present pattern and increasing the speed with which it is carried out.

There were differences in sequence-specific learning between the two groups. While both groups saw significant improvements in response time for both the Random and Repeated sequences, the Left Arm group saw greater improvements in the Random sequence compared to the Repeated sequence while the Right Arm group saw similar degrees of improvement in both sequences. The larger improvement in the Random sequence in the Left Arm group may have been related to the longer responses time for this condition at baseline. This difference in changes with practice between the two groups may also align with the idea that the dominant arm controller is more adept at predictive control of movements while the non-dominant arm controller is more adept at reacting to unpredictable or unfamiliar task conditions (Kitchen et al. 2024). The dominant arm controller’s specialty for predictive movements and task conditions may also be reflected in the Right Arm group having a greater reported awareness of the Repeated sequence after the completion of all practice compared to the Left Arm group (Baird and Stewart 2018) indicating a focus on predictive control of the repetitive sequential movements regardless of the task’s implicit learning design.

Understanding the differences in learning strategies for the dominant and non-dominant arms is important for rehabilitation. Conditions like stroke, Parkinson’s, and multiple sclerosis can affect movement control in both upper extremities. Knowledge about how movement is controlled and learned in young, healthy populations can help to better identify how arm function and learning is affected by these conditions which could, in turn, lead to more effective treatment practices. For example, after stroke, differences in reach control have been shown between the right and left arms, even when the moving arm is ipsilateral to the stroke (or less affected by the stroke) (Schaefer et al. 2007, 2009; Stewart et al. 2014); the ipsilesional arm may be a target for intervention in some individuals post-stroke (Sainburg and Duff 2006; Maenza et al. 2021). Further investigation comparing learning in the two arms could yield greater knowledge about how they adapt their motor patterns over practice and inform clinicians how to better approach rehabilitation protocols. Furthermore, knowledge of skill learning using the non-dominant limb may also be of importance for amputee populations or other populations who have lost function of their dominant limb as they will likely need to adapt to using the non-dominant limb to complete everyday tasks instead.

The sequences used in this study involved multi-directional, targeted reaching that simulate functional movements used in everyday life but were balanced for difficulty only based upon total inter-target distance and Fitts’ ID. Previous studies have shown that reach direction has an impact on outcomes such as movement time, peak velocity, and inter-joint coordination patterns such that some reaches may be easier than others based upon their direction (Gordon et al. 1994a, b; Dounskaia et al. 2002; Dounskaia 2005). While this would present a particular problem for the Random sequences, similar changes over practice were seen in the Repeated sequence which remained constant throughout practice. Regardless of the potential effects imparted by variances in directional combinations between the Random and Repeated sequences, learning occurred for both sequences in both arms. Similar to the previous study which examined the Right Arm group alone, the present study used hand path data from a single sensor on the fingertip to infer changes in inter-joint coordination (Baird and Stewart 2018). The use of multiple sensors during target-based reaching has provided information on the dynamics and coordination of multi-jointed reaching movements (Sainburg and Kalakanis 2000; Sainburg and Schaefer 2004; Dounskaia and Wang 2014; Yadav and Sainburg 2014), however, these previous studies have largely examined coordination using 2D reaching movements. Future studies examining sequence learning during 3D arm movements could include multiple sensors to allow examination of changes in inter-joint coordination in the right and left arms with practice. Because this study required end-point accuracy as a necessity to task completion and performance (i.e. had to capture the target for the next target to appear), it differs from other previous sequence learning studies using targeted reaching in which accuracy was emphasized and measured but capturing the target was not required for task progression (Ghilardi et al. 2009; Moisello et al. 2009). However, the inclusion of end-point accuracy as imperative to performance is important and adds to the applicability of results to real-world function as many functional tasks of daily living require end-point accuracy for successful completion.

In conclusion, both the dominant right and non-dominant left arms learned an implicit, multi-directional, targeted reaching sequence task, however, the approach taken to improve response time differed between arms. The dominant right arm improved performance through gains in both spatial control (shorter path distances) and movement speed (increased movement velocity) while the non-dominant left arm improved performance primarily through improvements in spatial control. Differences in the approach to improving performance between the two arms may be related to baseline differences in reach control. These results can be used as a model for identifying differences in reach control and sequence learning using whole-arm movements in clinical populations with movement disorders.

## Electronic supplementary material

Below is the link to the electronic supplementary material.

Supplementary Material 1

## Data Availability

The datasets generated during and/or analyzed in the current study are available from the corresponding author on reasonable request.
