# Supplementary material for: Implicit motor sequence learning using three-dimensional reaching movements with the non-dominant left arm
Source: Exp Brain Res. 2024 Oct 8;242(12):2715–26. doi: 10.1007/s00221-024-06934-4 (PMC11569025; doi:10.1007/s00221-024-06934-4)
Supplement: Supplementary file 1 — Supplementary Material 1 [file 221_2024_6934_MOESM1_ESM.docx]

**Supplemental Data**

**Implicit Motor Sequence Learning Using Three-Dimensional**

**Reaching Movements with the Non-Dominant Left Arm**

Charles R Smith^1^, Jessica F Baird^2^, Joelle Buitendorp^1^, Hannah Horton^1^,

Macie Watkins^1^, and Jill C Stewart^1§^

*^1^Department of Exercise Science, Arnold School of Public Health, University of South Carolina, Columbia SC*

*^2^Johns Hopkins Trial Innovation Center, Johns Hopkins School of Medicine, Baltimore, MD*

§Corresponding Author:

Jill Campbell Stewart, PT, PhD

Email: [jcstewar@mailbox.sc.edu](mailto:jcstewar@mailbox.sc.edu)

Phone: 803-777-6583

**Supplemental Results**

*Exploratory Analysis of Block 1*

A 2 X 2 X 9 (Group X Sequence X Repetition) mixed model ANOVA was completed to explore the first block of practice (Block 1, first 9 repetitions of each sequence). Significant effects for Repetition were found for Response Time (p < 0.001, η^2^ = 0.290), Total Hand Path Distance (p < 0.001, η^2^ = 0.298), and Peak Velocity (p = 0.004, η^2^ = 0.171) which indicated that, overall, response times decreased, hand path distance decreased, and movement speed increased during the first block of practice (see Supplemental Figure 1). Overall, these results suggest that practice related improvements in performance started early in practice on Day 1. Significant effects for Sequence were also found for Response Time (p<0.001, η^2^ = 0.616) and Total Hand Path Distance (p<0.001, η^2^ = 0.611) but not for Peak Velocity (p = 0.520, η^2^ = 0.018). Follow-up paired t-tests evaluated the difference between the Random and Repeated sequences at each repetition for each group separately to examine when those sequence-specific differences were present. For the Right Arm group, response time for the Repeated sequence was faster than for the Random sequence at repetition 8 (p=0.001). For the Left Arm group, response time for the Repeated sequence was faster than for the Random sequence at repetition 3 (p=0.031) and repetition 5 (p = 0.015). Significant effects for Group were found for Total Hand Path Distance (p = 0.002, η^2^ = 0.355) and Peak Velocity (p = 0.015, η^2^ = 0.230) but not for Response Time (p = 0.059, η^2^ = 0.147). The Left Arm group had overall longer hand paths (mean difference = 29.051 cm) and higher movement speeds (mean difference = 10.127 cm/sec) compared to the Right Arm group, however, while the Left Arm group had longer response times, the two groups did not significantly differ in overall response time (mean difference = +2.828 sec).

**Supplemental Figures**

Supplemental Figure 1. Block 1 Task Performance


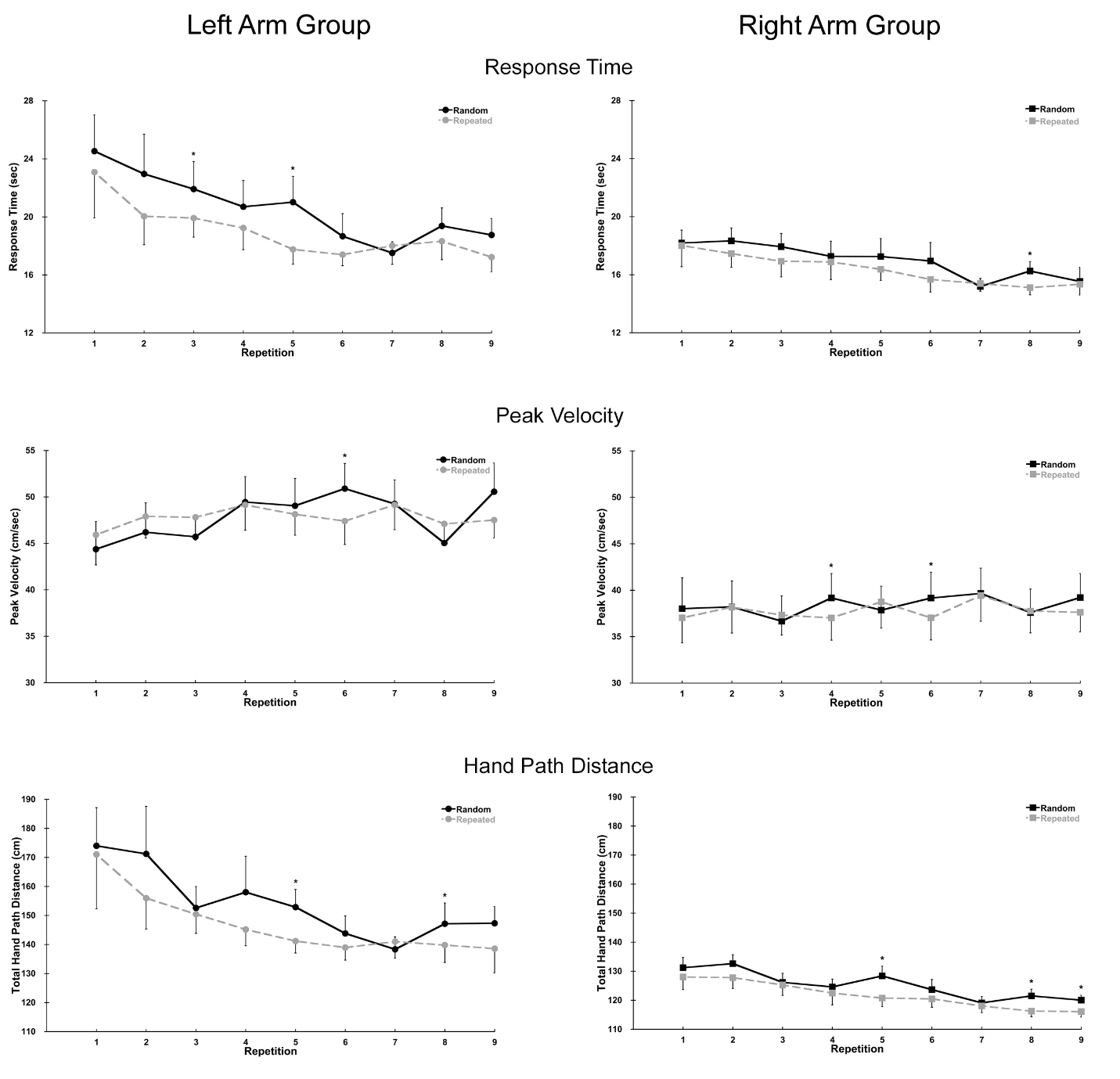


Supplemental Figure 1. Task performance for individual sequence trials in the first block of practice for the Right Arm and Left Arm Groups separately for response time (top row), peak velocity (middle row), and hand path distance (bottom row). Each repetition on the X axis represents performance for a single sequence repetition. Data presented as mean±SEM. *p<0.05 for difference between Random and Repeated sequence.

Supplemental Figure 2. Change in Task Performance from End of Day 1 to the Start of Day 2


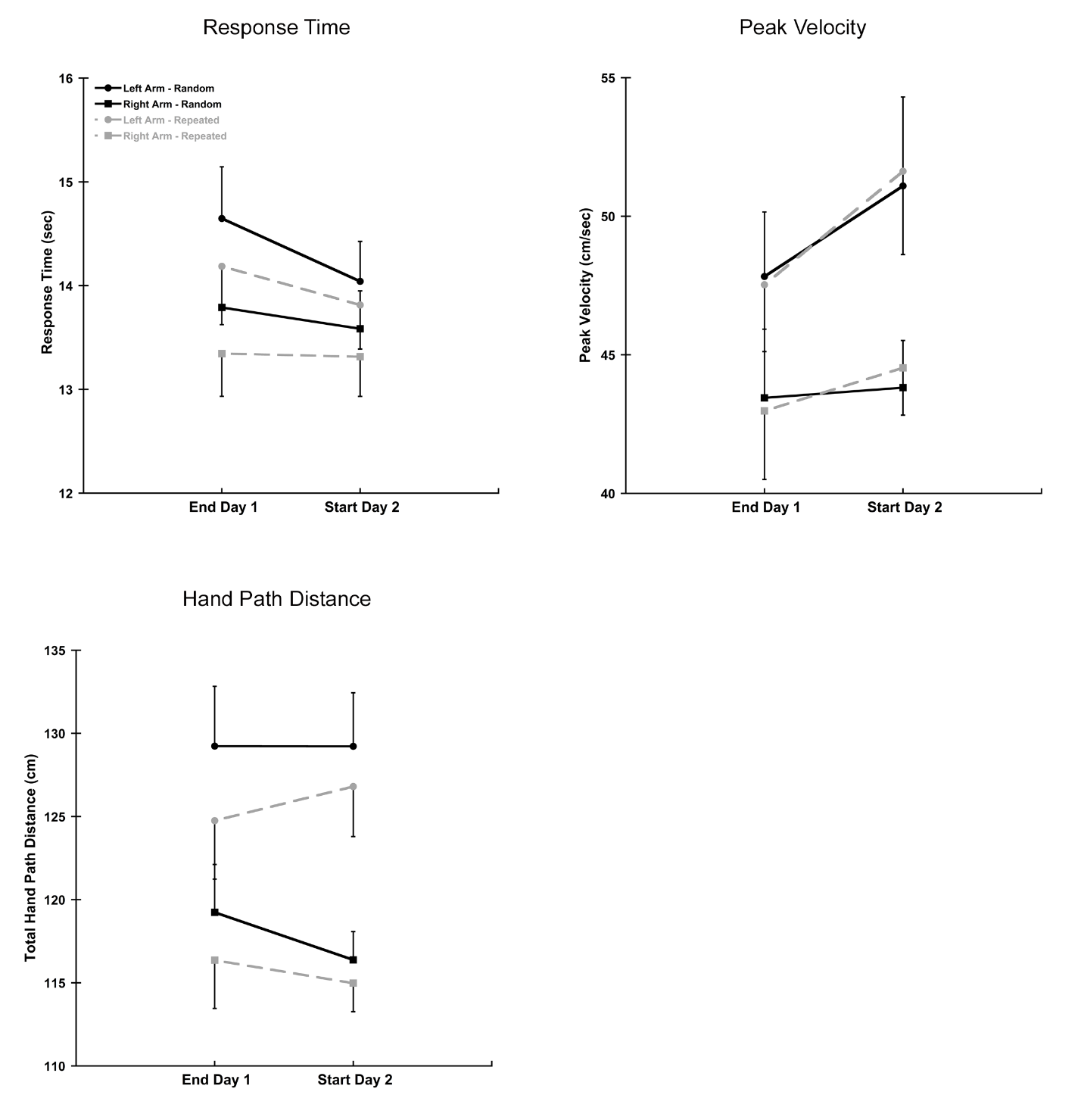


Supplemental Figure 2. Average response time, peak velocity, and hand path distance from the end of Day 1 (Block 8) to the start of Day 2 (Block 9) for Random (solid lines) and Repeated (dashed lines) sequences for each group. Data presented as mean±SEM.

**Supplemental Tables**

Supplemental Table 1. Statistical Summary for Baseline Performance

| **Effect** | ***df*** | ***F*** | ***p*-value** | **η^2^** |
| --- | --- | --- | --- | --- |
| **Response Time** |  |  |  |  |
| *Sequence* | (1, 29) | **26.972** | **<0.001** | **0.482** |
| *Group (Arm)* | (1, 29) | **4.327** | **0.046** | **0.130** |
| *Sequence X Group* | (1 ,29) | **5.886** | **0.022** | **0.169** |
|  |  |  |  |  |
| **Total Hand Path Distance** |  |  |  |  |
| *Sequence* | (1, 29) | **36.859** | **<0.001** | **0.560** |
| *Group (Arm)* | (1, 29) | **13.392** | **0.001** | **0.316** |
| *Sequence X Group* | (1 ,29) | 3.911 | 0.058 | 0.119 |
|  |  |  |  |  |
| **Peak Velocity** |  |  |  |  |
| *Sequence* | (1, 29) | 2.437 | 0.129 | 0.078 |
| *Group (Arm)* | (1, 29) | **9.382** | **0.005** | **0.244** |
| *Sequence X Group* | (1 ,29) | 0.130 | 0.721 | 0.004 |

Supplemental Table 2. Statistical Summary for Acquisition

| **Effect** | ***df*** | ***F*** | ***p*-value** | **η^2^** |
| --- | --- | --- | --- | --- |
| **Response Time** |  |  |  |  |
| *Block* | (7, 23) | **41.527** | **<0.001** | **0.589** |
| *Sequence* | (1, 29) | **82.687** | **<0.001** | **0.740** |
| *Group (Arm)* | (1, 29) | 1.975 | 0.171 | 0.064 |
| *Block X Sequence* | (7, 23) | **3.928** | **0.008** | **0.119** |
| *Block X Group* | (7, 23) | **4.177** | **0.026** | **0.126** |
| *Sequence X Group* | (1 ,29) | 0.059 | 0.810 | 0.002 |
| *Block X Sequence X Group* | (7, 23) | 2.532 | 0.054 | 0.080 |
|  |  |  |  |  |
| **Total Hand Path Distance** |  |  |  |  |
| *Block* | (7, 23) | **21.456** | **<0.001** | **0.425** |
| *Sequence* | (1, 29) | **73.122** | **<0.001** | **0.716** |
| *Group (Arm)* | (1, 29) | **10.012** | **0.004** | **0.257** |
| *Block X Sequence* | (7, 23) | **4.617** | **0.002** | **0.137** |
| *Block X Group* | (7, 23) | **6.849** | **0.002** | **0.191** |
| *Sequence X Group* | (1 ,29) | 0.048 | 0.827 | 0.002 |
| *Block X Sequence X Group* | (7, 23) | 2.080 | 0.086 | 0.067 |
|  |  |  |  |  |
| **Peak Velocity** |  |  |  |  |
| *Block* | (7, 23) | 2.217 | 0.110 | 0.071 |
| *Sequence* | (1, 29) | 0.160 | 0.692 | 0.005 |
| *Group (Arm)* | (1, 29) | **4.955** | **0.034** | **0.146** |
| *Block X Sequence* | (7, 23) | 2.447 | 0.050 | **0.427** |
| *Block X Group* | (7, 23) | **3.408** | **0.033** | **0.105** |
| *Sequence X Group* | (1 ,29) | 0.004 | 0.948 | <0.001 |
| *Block X Sequence X Group* | (7, 23) | 0.434 | 0.871 | 0.117 |

Supplemental Table 3. Statistical Summary for Retention

| **Effect** | ***df*** | ***F*** | ***p*-value** | **η^2^** |
| --- | --- | --- | --- | --- |
| **Response Time** |  |  |  |  |
| *Block* | (1, 29) | 2.792 | 0.105 | 0.088 |
| *Sequence* | (1, 29) | **27.631** | **<0.001** | **0.488** |
| *Group (Arm)* | (1, 29) | 1.975 | 0.171 | 0.064 |
| *Block X Sequence* | (1, 29) | 3.287 | 0.060 | 0.473 |
| *Block X Group* | (1, 29) | 1.046 | 0.315 | 0.035 |
| *Sequence X Group* | (1, 29) | 0.010 | 0.923 | <0.001 |
| *Block X Sequence X Group* | (1, 29) | 0.078 | 0.782 | 0.003 |
|  |  |  |  |  |
| **Total Hand Path Distance** |  |  |  |  |
| *Block* | (1, 29) | 0.152 | 0.699 | 0.005 |
| *Sequence* | (1, 29) | **67.827** | **<0.001** | **0.700** |
| *Group (Arm)* | (1, 29) | **7.717** | **0.009** | **0.210** |
| *Block X Sequence* | (1, 29) | **11.029** | **0.002** | **0.276** |
| *Block X Group* | (1, 29) | 1.247 | 0.273 | 0.041 |
| *Sequence X Group* | (1, 29) | 3.749 | 0.063 | 0.114 |
| *Block X Sequence X Group* | (1, 29) | 0.313 | 0.580 | 0.011 |
|  |  |  |  |  |
| **Peak Velocity** |  |  |  |  |
| *Block* | (1, 29) | **5.923** | **0.021** | **0.170** |
| *Sequence* | (1, 29) | 0.336 | 0.567 | 0.011 |
| *Group (Arm)* | (1, 29) | 2.315 | 0.139 | 0.074 |
| *Block X Sequence* | (1, 29) | **11.366** | **0.002** | **0.282** |
| *Block X Group* | (1, 29) | 2.031 | 0.165 | 0.065 |
| *Sequence X Group* | (1, 29) | <0.001 | 0.990 | <0.001 |
| *Block X Sequence X Group* | (1, 29) | 0.372 | 0.547 | 0.013 |
